# Proteomic Analysis of Aqueous Humor Proteins in Association with Cataract Risks: Diabetes and Smoking

**DOI:** 10.3390/jcm10245731

**Published:** 2021-12-07

**Authors:** Wei-Cheng Chang, Cho-Hao Lee, Shih-Hwa Chiou, Chen-Chung Liao, Chao-Wen Cheng

**Affiliations:** 1 Graduate Institute of Clinical Medicine, College of Medicine, Taipei Medical University, Taipei 11031, Taiwan; cwc761229@gmail.com; 2 Department of Ophthalmology, Taoyuan General Hospital, Ministry of Health and Welfare, Taoyuan 33004, Taiwan; 3 Division of Hematology and Oncology, Department of Internal Medicine, Tri-Service General Hospital, National Defense Medical Center, Taipei 114202, Taiwan; drleechohao@gmail.com; 4 Department of Medical Research, Taipei Veterans General Hospital, Taipei 11217, Taiwan; shchiou@vghtpe.gov.tw; 5 School of Medicine, National Yang-Ming University, Taipei 11221, Taiwan; 6 Institute of Pharmacology, National Yang-Ming University, Taipei 11221, Taiwan; 7 Genomic Research Center, Academia Sinica, Taipei 11529, Taiwan; 8 Metabolomics-Proteomics Research Center, National Yang Ming Chiao Tung University, Taipei 11221, Taiwan; ccliao@ym.edu.tw; 9 Traditional Herbal Medicine Research Center, Taipei Medical University Hospital, Taipei Medical University, Taipei 11031, Taiwan; 10 Cell Physiology and Molecular Image Research Center, Wan Fang Hospital, Taipei Medical University, Taipei 11031, Taiwan

**Keywords:** aqueous humor, label free, cataract, risk factor, proteomics, alpha-2-HS-glycoprotein, fetuin-A

## Abstract

Cataracts are one of the most common eye diseases that can cause blindness. Discovering susceptibility factors in the proteome that contribute to cataract development would be helpful in gaining new insights in the molecular mechanisms of the cataract process. We used label-free nanoflow ultra-high-performance liquid chromatography–tandem mass spectrometry to compare aqueous humor protein expressions in cataract patients with different cataract risk factors such as diabetes mellitus (DM) and smoking and in controls (with cataract) without risk exposure. Eight patients with diabetes and who smoked (with double risk factors), five patients with diabetes and five patients who smoked (both with a single risk factor), and nine aged-matched cataract controls patients (non-risk exposure) were enrolled. In total, 136 aqueous humor proteins were identified, of which only alpha-2-Heremans–Schmid (HS)-glycoprotein was considered to be significantly risk-associated because it was differentially expressed in these three groups and exhibited increased expression with increasing risk factors. Significant changes in the aqueous humor level of alpha-2-HS-glycoprotein between DM and control samples and between smoking and control samples were confirmed using ELISA. The alpha-2-HS-glycoprotein, called fetuin-a, could be a potential aqueous biomarker associated with DM and smoking, which were cataract risk factors.

## 1. Introduction

In developed countries, cataracts are one of the most common causes of blindness [1]. They are classified by cause as age-related cataracts, pediatric cataracts, and cataracts secondary to other causes. As shown by many studies, age is the biggest risk factor [2,3]. Considering the location of opacification within the lens, cataracts are divided into three major types: nuclear, cortical, and posterior subcapsular cataracts. Cataract development can be caused by many other risk factors, including environmental factors and genetic changes [4]. Diabetes mellitus (DM), long-term use of corticosteroids, cigarette smoking, prolonged exposure to ultraviolet light, and alcohol abuse are well-known risk factors [2]. Cigarette smoking is a risk factor for nuclear and posterior subcapsular cataracts [4]. DM was identified as a common cause of posterior subcapsular and cortical cataracts [5,6]. Increased age is a risk factor for the development of all types of cataracts. Throughout life, a high myopia of over −6.0 D is associated with nuclear cataracts and posterior subcapsular cataracts [7]. Other causes of cataracts include mechanical trauma, chemical injury, electrical injury, radiation, and certain medications. However, the underlying cataractogenic mechanisms of cataract development are still not well documented, with many still being investigated. Proteomics analysis is an extensively used technique to discover changes in protein levels in tissues and cells. Recent proteomic studies in cataract disease of the human aqueous humor (AH) revealed multiple proteins of interest in patients [8,9,10,11,12]. Ji et al. [13] used isobaric tags for the relative and absolute quantitation (iTRAQ) methodology to compare AH protein profiles among high myopia, glaucoma, and vitrectomy surgery patients, and controls. They identified multiple candidate protein biomarkers associated with cataract development in each group. Furthermore, Kim et al. [14] analyzed the aqueous proteome from age-related macular degeneration (AMD) patients and non-AMD cataract controls to identify novel pathogenic proteins that are useful as potential clinical biomarkers. The differential expressions of three proteins were reported in the AH of AMD patients compared with those of cataract controls. Those studies used a good model that inspired a new idea for us of using proteomics to discuss different risk factors of cataract formation. To our knowledge, there has been no previous investigation of different cataract risk factors by comparing proteomic evidence. We used proteomics to discover the pathogenesis of different cataract risks and to possibly identify candidate biomarker proteins identified in patients predisposed to this condition. In this study, we employed Nanoflow ultra-high-performance liquid chromatography–tandem mass spectrometry (n-UPLC-MS/MS) to examine the protein compositions of aqueous solutions obtained from human cataract eyes of patients who had a single risk factor of either DM or cigarette smoking, those who had double risk factors of DM and cigarette smoking, and aged-matched cataract controls (with neither risk factor). This sensitive proteomics approach could help examine the underlying pathophysiology of cataract formation using relatively scarce amounts of aqueous samples, thereby favoring the methodological approach for this investigation. This study may reveal valuable insights into the molecular changes in the AH in the course of cataract pathogenesis.

## 2. Materials and Methods

### 2.1. Subjects

The study protocol was approved by the Medical Ethics and Institutional Review Board of Taoyuan General Hospital, Ministry of Health and Welfare (TYGH109009) (Taoyuan, Taiwan), and conducted as per the tenets of the Declaration of Helsinki. All study participants provided written informed consent before their enrollment, and the nature and possible consequences of the study were explained to them. Human AH samples from treatment-naive patients with a single risk factor (*n* = 10) of DM (*n* = 5) or cigarette smoking (*n* = 5), double risk factors (*n* = 8) of DM combined with cigarette smoking, and aged-matched cataract controls with neither risk factor (*n* = 9) were collected while patients were undergoing cataract surgery at Taoyuan General Hospital. The diagnostic criterion for cataracts was defined with a slit lamp with no other ocular diseases, trauma, or previous intraocular operation history. The presence of type 2 diabetes was defined as any one or more of the following: (1) having had a diagnosis of type 2 diabetes that was confirmed by a physician (ICD10: E11); (2) self-report of a diabetes diagnosis and use of hypoglycemic medications; (3) a fasting glucose level of ≥126 mg/dL; (4) a 2 h post-challenge plasma glucose level of ≥200 mg/dL. All subjects were included as cases of type 2 diabetes within a follow-up time of five years. A cigarette smoking history was obtained from all patients. Their cigarette consumption varied with a mean duration of more than 20 years. Data on control eyes were collected from senior cataract patients who were free from other ocular or systemic diseases. In these three groups, inclusion criteria were cataract patients aged older than 55 years. Exclusion criteria were a history of any systemic or ocular disorder or condition including ocular surgery, trauma, or disease. Best corrected visual acuity (BCVA) was measured as the logarithm of the minimum angle of resolution (logMAR).

### 2.2. AH Sample Collection

AH samples were obtained from patients during the implantation of phakic intraocular lenses. To avoid hemorrhaging and ocular surface contamination, a sample was collected using a 1 mL tuberculin syringe with a 30 gauge needle at the limbus before any other entry into the eye under a surgical microscope. Note that 50–100 μL of AH was collected from each patient by anterior chamber paracentesis. Undiluted AH samples were collected and stored at −80 °C within 24 h until preparation was initiated.

### 2.3. n-UPLC-MS/MS

**Figure 1:**
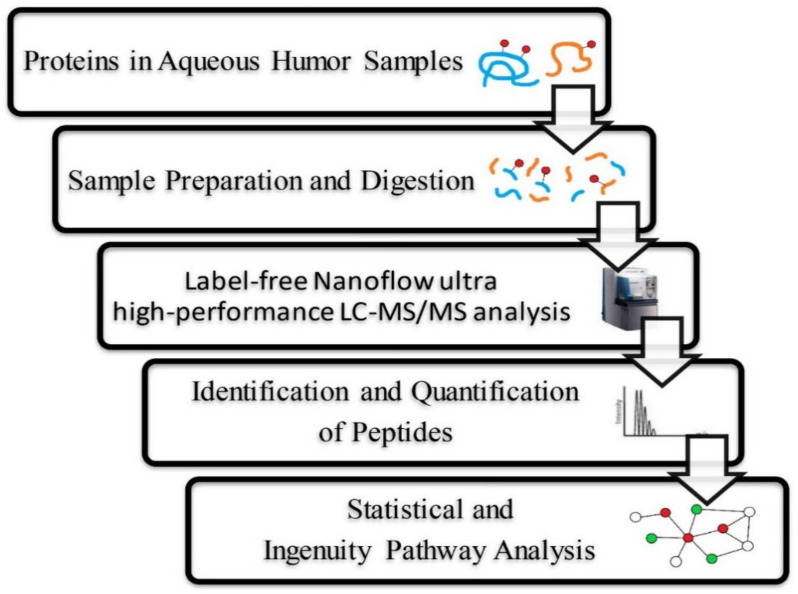
Label-free Nanoflow UHPLC-MS/MS analytical workflow for the proteomic analysis of human aqueous humor. Samples were digested using trypsin and were analyzed using an LTQ-Orbitrap DiscoveryTM hybrid mass spectrometer (Thermo Electron). Proteins were identified and quantified using the SEQUEST algorithm followed by analysis using Xcalibur 2.0 SR1 (Thermo Electron).

Protein concentrations of AH samples were determined by a dye-binding method based on the Bradford assay (Bio-Rad Laboratories, Richmond, CA, USA) (Table 1), and samples were further diluted in 1× phosphate-buffered saline (PBS) to a final concentration of 0.1 μg/μL. Samples were prepared as per the SMART digestion kit protocol from ThermoFisher Scientific (Waltham, MA, USA) and cleaned up using solid-phase extraction (SPE) plates from ThermoFisher. The resulting peptides collected from the filters were dried in a vacuum centrifuge and stored at −80 °C. Then, 50 μL of diluted AH samples was resuspended in 0.1% formic acid and analyzed by n-UPLC-MS/MS. Tryptic peptides were loaded into an LTQ-Orbitrap mass spectrometer with a nanoelectrospray ionization source (Thermo Electron, MA, USA) connected to a nanoACQUITY UPLC system (Waters, MA, USA). Peptide samples were separated on a 25 cm × 75 μm BEH130 C18 column (Waters) with a 0–95% segmented gradient of 3–40% B for 168 min, 40–95% B for 2 min, and 95% B for 10 min at a flow rate of 0.5 μL/min. Mobile phase A was 0.1% formic acid in water, while mobile phase B was 0.1% formic acid in acetonitrile. The mass spectrometer was set to the data-dependent acquisition method (isolation width: 1.5 Da). As per the data-dependent acquisition method, the first ten most intensively charged peptide ions were selected and fragmented using a collision-induced dissociation (CID) method (Figure 1).

### 2.4. Protein Identification

Then, the acquired MS/MS raw data files were applied to search against a UniProt human protein database (containing 20,387 protein sequences; released on 9 April 2021; http://www.uniprot.org/ (accessed on 6 December 2021)) with PEAKS Studio 7.5 (Bioinformatic Solution, Ontario, CA, USA). The search settings of PEAKS Studio 7.5 combined with UniProt’s protein database were as follows: enzyme set to trypsin; up to two missing cut sites; precursor and fragment mass tolerances of 20 ppm and 0.8 Da, respectively; false discovery rate (FDR) of <1%, obtained from a search of the decoy database. Furthermore, based on a label-free quantitative analysis, each identified protein had to contain at least one unique peptide and protein quantification method. Moreover, spectral counts were normalized to the total identification spectrum of each biological sample.

### 2.5. Enzyme-Linked Immunosorbent Assay (ELISA)

An alpha-2-Heremans–Schmid (HS)-glycoprotein ELISA assay was performed to measure concentrations of AH samples among the single-risk group, double-risk group, and the age-matched cataract controls with a Human Alpha-2-HS-glycoprotein ELISA Kit (EH310RB, ThermoFisher Scientific), as per the manufacturer’s protocol.

### 2.6. Statistical Analysis

Clinical data were analyzed using Stata (vers. 16.1, StataCorp, College Station, TX, USA) to define the statistical significance between groups by a *t*-test or Chi-squared test, and *p* < 0.05 was considered to be statistically significant. Statistical analysis by Fisher’s exact test, Wilcoxon test, or Kruskal–Wallis test was used to confirm that there were no statistically significant differences in age among the single-risk group, double-risk group, and the age-matched cataract control group (Table 1).

Note: Single risk, patients with the DM or smoking risk factor; double risk, patients with both the DM and smoking risk factors; control, cataract patients with neither of these cataract risk factors; VA, visual acuity; AXL, axial length.

## 3. Results

**Figure 2:**
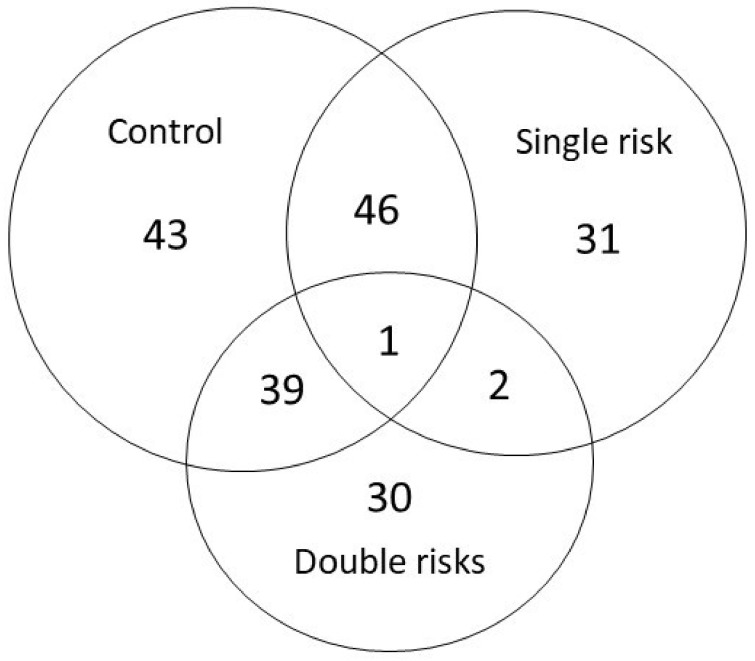
Label-free Nanoflow UHPLC-MS/MS analytical workflow for the proteomic analysis of human aqueous humor. Samples were digested using trypsin and were analyzed using an LTQ-Orbitrap DiscoveryTM hybrid mass spectrometer (Thermo Electron). Proteins were identified and quantified using the SEQUEST algorithm followed by analysis using Xcalibur 2.0 SR1 (Thermo Electron). The intersection of each area represents the number of significant expression (*p* < 0.05) proteins between each groups. Only one protein was significantly deferentially expressed in each group.

Table 1 lists the demographic data of patients with a single risk factor, those with double risk factors, and the control group (with cataract). The mean age of single-risk-factor patients was 72.30 ± 10.14 years, for double-risk-factor patients was 69.38 ± 9.87 years, and for cataract control individuals was 74.00 ± 5.72 years. All patients had cataracts as revealed by a slit lamp examination. The mean protein concentrations were 0.36 ± 0.21 μg/μL in the single-risk-factor group, 0.34 ± 0.11μg/μL in the double-risk-factor group, and 0.22 ± 0.06 μg/μL in the cataract control group. There were statistical differences among total protein contents in these three groups (*p* = 0.049) but no statistical differences in age among these groups (*p* = 0.390). In total, 136 proteins were successfully identified by LC-ESI MS/MS in single-risk-factor, double-risk-factor, and cataract control AH samples (Table 2, Figure 2).

**Figure 3:**
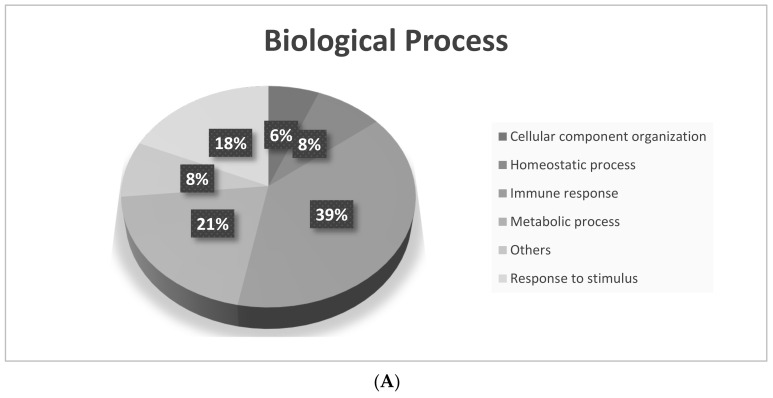

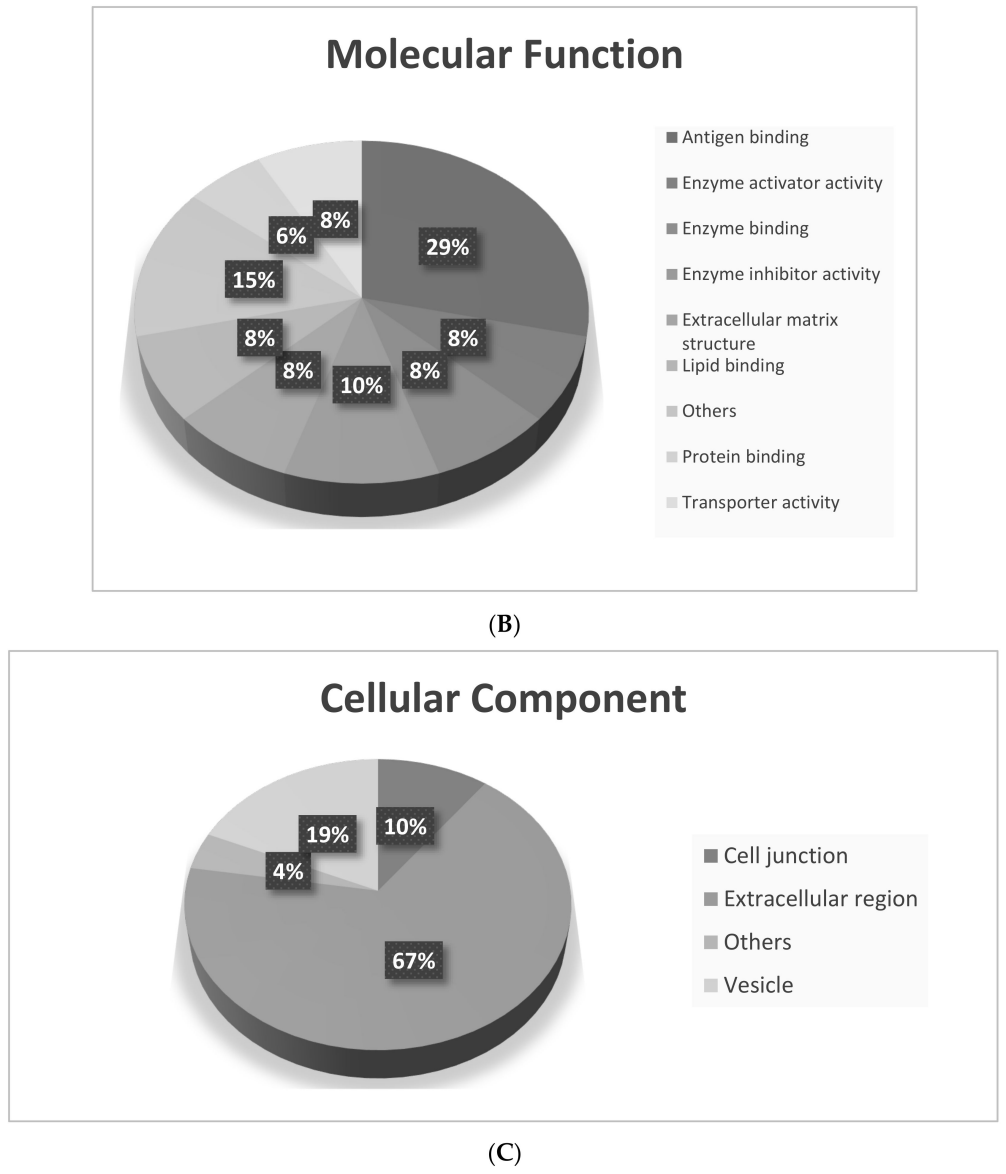
Gene ontology (GO) analysis of differentially expressed proteins of the aqueous humor (AH) in the cataract control, single-risk, and double-risk groups. We compared identified AH proteins from the three groups: (**A**) biological processes; (**B**) molecular functions; (**C**) cellular components.

Comparing the single-risk group to the cataract control group, 125 proteins were found, which included 42 proteins that were present at higher expression levels and 83 proteins that were present at lower expression levels in the single-risk group. In the double-risk group, as compared to the cataract control group, 124 proteins were disclosed, among which 39 proteins had higher expression levels and 85 proteins had lower expression levels in the double-risk group. To understand the biological meaning of the changes of protein expression observed in different risk factor groups, differentially expressed proteins were analyzed for “molecular functions”, “biological processes”, and “cellular components” by GO annotations. Our results demonstrated that differentially expressed proteins in the three groups had different molecular functions, biological processes, and cellular components (Figure 3). The major biological processes of these proteins were biological regulation, including immune responses, metabolic processes, and responses to stimuli of the AH (Figure 3A). The major molecular functions of AH proteins enriched among single-risk and double-risk patients were antigen binding and enzyme inhibitor activity (Figure 3B). As per cellular component terms of the GO, most significant AH proteins were categorized as extracellular region proteins (Figure 3C). Then, we used Ingenuity Pathway Analysis (IPA, Qiagen) to show canonical pathways that are potentially involved in the pathogenesis of cataracts under the risks of diabetes and smoking. Table 3 lists pathways associated with AH proteins from single-risk patients, double-risk patents, and the cataract controls.

The top canonical pathways, including LXR/RXR activation, FXR/RXR activation, and acute-phase response signaling, demonstrated significant associations with AH proteins. Statistical analysis was performed on these 136 proteins. In total, 47 proteins exhibited statistically significant changes in content in the group with a single risk factor compared to the cataract control group (Table 4).

**Figure 4:**
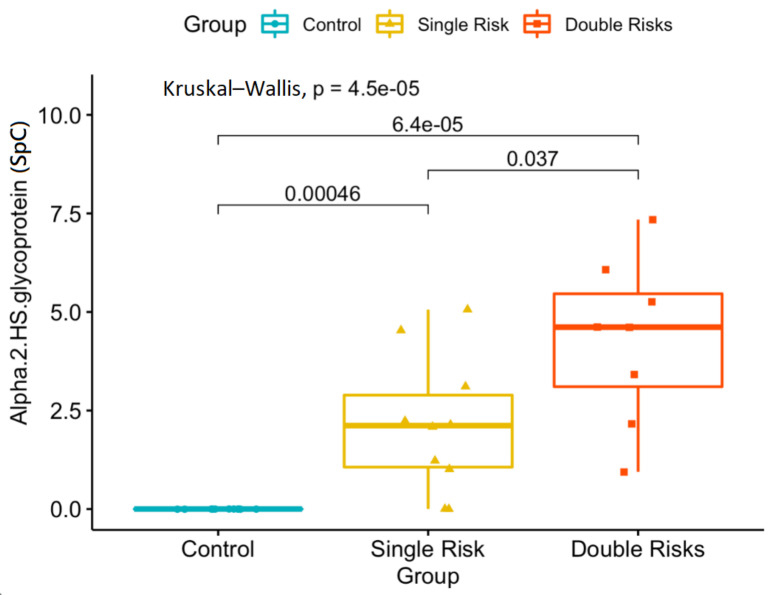
Proteomics analysis revealed significant concentration changes in the alpha-2-HS-glycoprotein (SpC, spectral count) among the three groups. Single risk, patients with the diabetes mellitus (DM) or smoking risk factor; double risk, patients with both the DM and smoking risk factors; control, cataract patients with neither of these cataract risk factors.

In a comparison of the double-risk-factor group with the cataract control group, 40 proteins were statistically significantly (*p* < 0.05) expressed (Table 4). Among the 51 proteins that were significantly changed, 10 proteins were increased in the single- or double-risk groups, including 26S proteasome non-ATPase regulatory subunit 1, alpha-2-HS-glycoprotein, apolipoprotein A-I, apolipoprotein A-II, apolipoprotein A-IV, apolipoprotein E, opticin, potassium voltage-gated channel subfamily S member 2, complement C4-A, and complement C4-B. Another 41 proteins exhibited decreased expression in the single- or double-risk groups compared to cataract controls (Table 4). In particular, alpha-2-HS-glycoprotein was the only one that presented a significant change among all three of the groups (cataract control vs. single: *p* = 0.00338; cataract control vs. double: *p* = 0.00062; single vs. double: *p* = 0.03309), which demonstrated an increasing trend with increase in risk (Figure 4).

**Figure 5:**
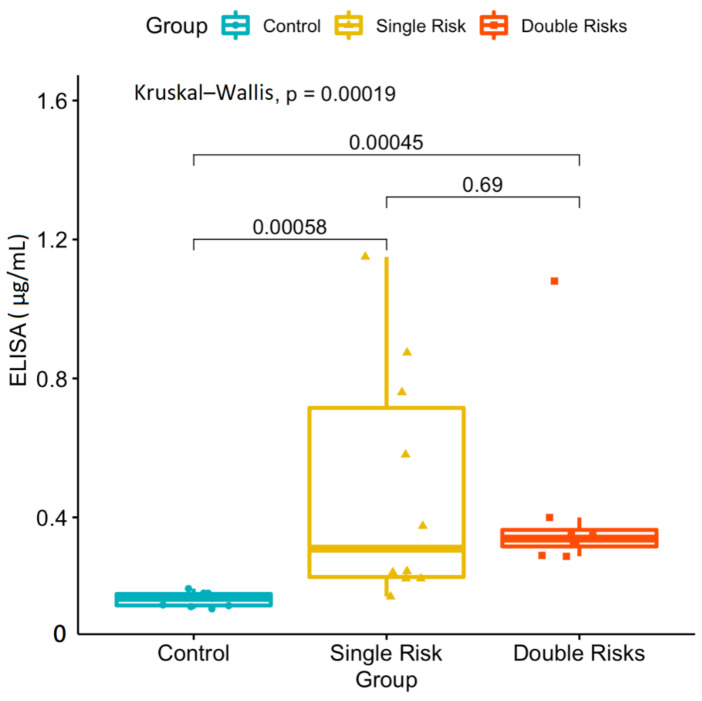
ELISA analysis of significant concentration (μg/mL) changes of the alpha-2-HS-glycoprotein between risk factor and cataract control groups. However, there was no significant concentration change between the single- and double-risk-factor groups. Single risk, patients with the diabetes mellitus (DM) or smoking risk factor; double risk, patients with both the DM and smoking risk factors; control, cataract patients with neither of these cataract risk factors.

Furthermore, we performed an ELISA analysis to determine the concentration of alpha-2-HS-glycoprotein. Compared to the cataract control group, the average concentration of alpha-2-HS-glycoprotein was significantly higher in single-risk-factor group (0.43 μg/mL) patients (0.16 μg/mL) (*p* = 0.002) (Figure 5).

**Figure 6:**
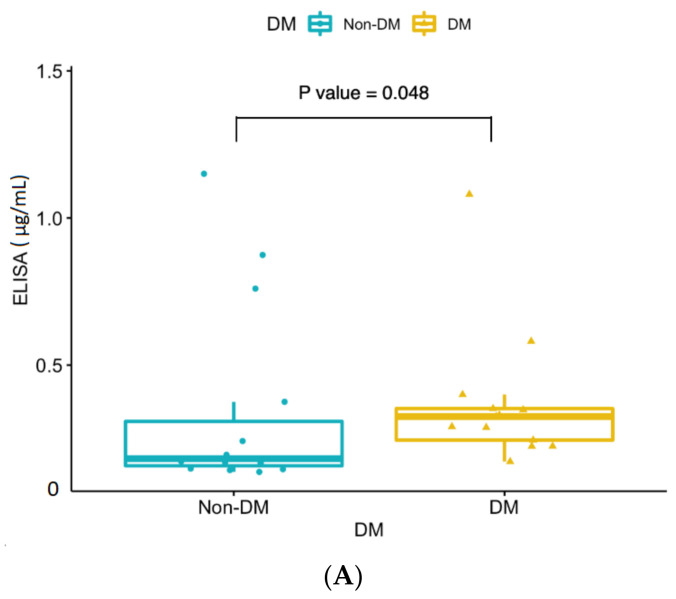

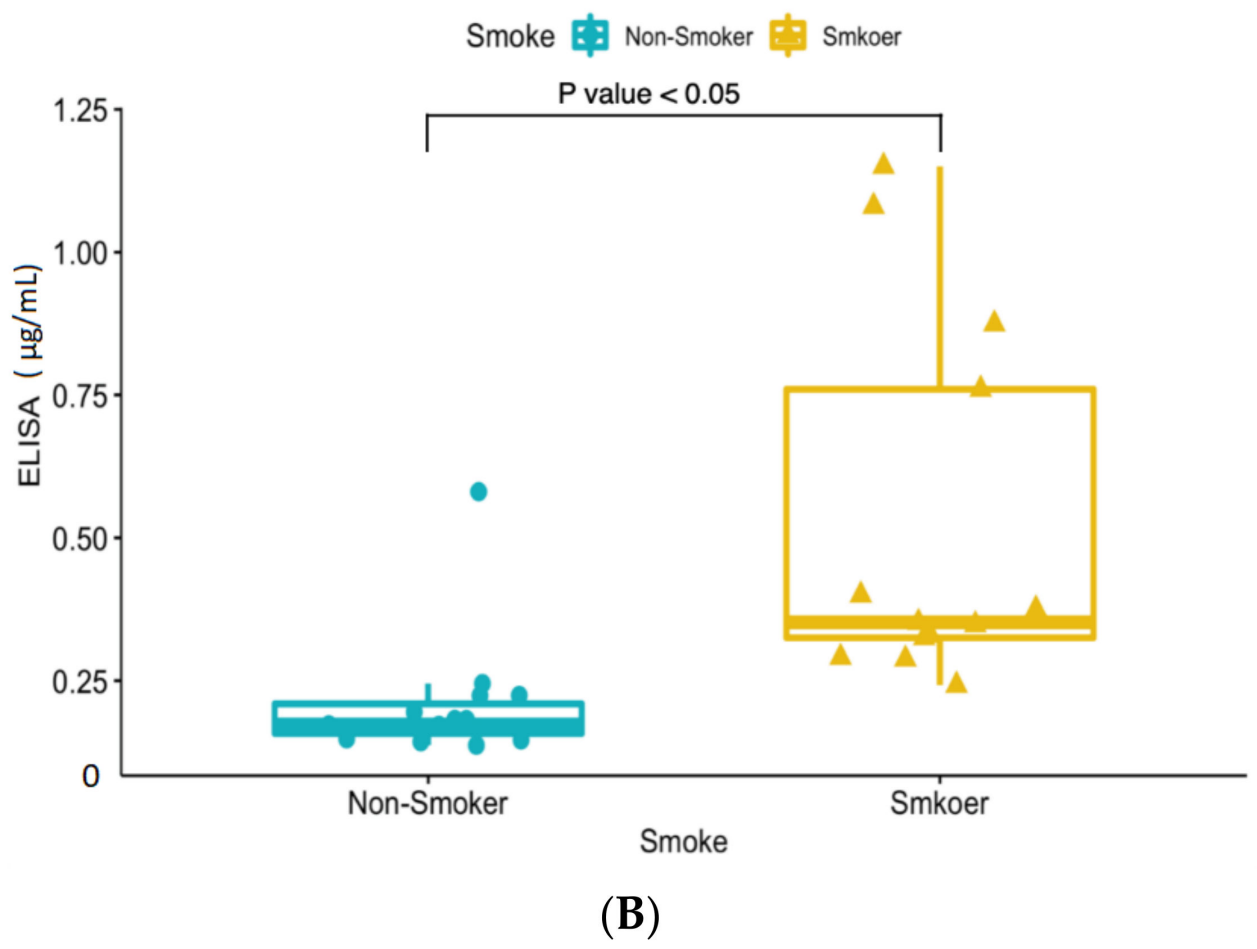
(**A**) ELISA analysis of significant concentration (μg/mL) changes in the alpha-2-HS-glycoprotein between the diabetes mellitus (DM) groups and cataract control group. (**B**) ELISA analysis of significant concentration (μg/mL) changes in the alpha-2-HS-glycoprotein between the smoking groups and cataract control group. DM group (*n* = 13): DM single-risk patients (*n* = 5) + double-risk patients (*n* = 8); Non-DM group (*n* = 14): smoking single-risk patients (*n* = 5) + cataract control group (*n* = 9); Smokers (*n* = 13): smoking single-risk patients (*n* = 5) + double-risk patients (*n* = 8); Non-smokers (*n* = 14): DM single-risk patients (*n* = 5) + cataract control group (*n* = 9).

Furthermore, the average concentration significantly increased in double-risk-factor group (0.43 μg/mL) patients compared to the cataract control group (0.16 μg/mL) (*p* < 0.001) (Figure 5). The ELISA analysis revealed significant concentration changes between the risk factor and cataract control groups. However, there was no significant concentration change between the single- and double-risk-factor groups. A subgroup analysis was performed to confirm that DM and smoking risk factors significantly influenced the ELISA concentration compared to the cataract control group (Figure 6).

In our study, we analyzed the aqueous protein contents of the AH samples of single-risk and double-risk patients and a control group (with cataract) using label-free n-UPLC-MS/MS quantitation. We reported that in cataract patients with different risk profiles, 51 AH proteins were significantly changed compared to cataract controls. The alpha-2-HS-glycoprotein was significantly differently expressed between risk groups and cataract controls and could be a potential aqueous protein marker for detecting smoking and DM cataract risk factors. The increased levels of total protein concentrations were reported in the AH, which provides a possible marker to monitor the AH of cataract risk exposure. Note that additional studies exploring the roles of this protein in the development or the pathogenesis molecular pathway of cataracts would be beneficial. To our knowledge, this is the first study to analyze how cataract risk factors influenced the AH in the development of cataract disease. We reported that only one protein had significantly changed, which was the alpha-2-HS-glycoprotein; its expression increased in the presence of risk factors. Alpha-2-HS-glycoprotein, known as fetuin-A, was reported to be a systemic inhibitor of precipitation of basic calcium phosphate, thereby preventing unwanted calcification [15] and influencing the mineral phase of bone [16]. The alpha-2-HS-glycoprotein is synthesized in the liver, electively concentrated in the bone matrix, and secreted in plasma. The dysfunction of the gene represented by this entry is associated with alopecia-mental retardation syndrome [17]. There was previous evidence demonstrating that the alpha-2-HS-glycoprotein was present in the rabbit AH following two different cataract surgery incision procedures [18]; furthermore, there were significant decreases in the AH of 5-year-old buphthalmic rabbits [19] but not in the 2-year-old group, demonstrating that alpha-2-HS-glycoprotein alters with pathologic changes in DM, anterior lens capsule, and the angular meshwork. In humans, it was shown to be an inhibitor of transforming growth factor (TGF)-β2 [20], a protein that shows increased expression in the trabecular meshwork (TM) in open-angle glaucoma causing extracellular matrix (ECM) deposition in the human TM [21]. The alpha-2-HS-glycoprotein inhibits bone morphogenetic proteins that are changed in the TM in open-angle glaucoma [22]. This evidence suggests the potential interactions of the alpha-2-HS-glycoprotein with multiple proteins that are important in open-angle glaucoma. However, there is scarce evidence demonstrating a relationship between the alpha-2-HS-glycoprotein and cataract disease in human beings to date. Interestingly, the serum levels of alpha-2-HS-glycoprotein, called fetuin-A, are known to be highly associated with DM in humans. Initially, it was discussed in the context of preventing glucose toxicity in early 2002 [23,24]. Then, in the past two decades, the alpha-2-HS-glycoprotein was linked to insulin resistance, obesity, and cardiovascular diseases [25,26,27,28,29,30,31]. Guo et al. and Roshanzamir et al. revealed evidence using meta-analyses that higher serum alpha-2-HS-glycoprotein levels are associated with increased risk of type 2 DM [32,33]. All these previous studies reported the correlation of alpha-2-HS-glycoprotein levels in urine [34] or serum [35] with diabetes. Yuksel et al. performed a serum and AH alpha-2-HS-glycoprotein (fetuin-A) level comparison in pseudoexfoliation syndrome (PEXS) patients [36]. They found significantly increased alpha-2-HS-glycoprotein levels in the AH of patients with PEXS, but no correlation between the AH and serum levels of alpha-2-HS-glycoprotein between the groups. They suggested that the increase in alpha-2-HS-glycoprotein levels in the AH was due to disruption of the blood–aqueous barrier because of the hypoperfusion and anterior chamber hypoxia in PEXS. Thus, until now there was scarce evidence to prove that the serum level of alpha-2-HS-glycoprotein was associated with that in AH. However, our results are the first to report that human aqueous levels of the alpha-2-HS-glycoprotein are associated with diabetes risk factors for cataract formation. The ELISA confirmation of aqueous alpha-2-HS-glycoprotein levels confirmed these results. In certain diabetic patients, we provide a novel way of thinking about changes in alpha-2-HS-glycoprotein levels in the circulation and in the aqueous fluid. We suggest that the alpha-2-HS-glycoprotein could be an aqueous-specific marker of cataract risk, which is highly associated with diabetes. The alpha-2-HS-glycoprotein is known as an immune-reactive protein that was determined to be smoking- and age-associated with the development of head and neck cancers. The consistent association of chronic smoking shows an immune reactivity status that changes the serum levels of alpha-2-HS-glycoprotein in head and neck cancer patients [37]. Marechal et al. demonstrated a negative correlation between serum fetuin-A levels and a history of smoking, in which fetuin-A levels were determined by a common haplotype of the *AHSG* gene, low plasma cholesterol, and a history of smoking in renal transplant recipients [38]. They considered that it might reflect consequences of tobacco smoking on liver function, physical activity, or weight loss, which increased aortic calcification and risk of cardiovascular events in renal transplant recipients. These previous studies support our result that the alpha-2-HS-glycoprotein may be associated with the smoking habit. We considered that the alpha-2-HS-glycoprotein could be an aqueous-specific marker of cataract risks that is highly associated with smoking. However, multiple limitations of this study should be reported. First, only eight to ten samples in each group were investigated, and future large-scale studies could help confirm our results. The small sample numbers may be attributed to ELISA, which could not validate the proportional results of aqueous alpha-2-HS-glycoprotein levels in the three groups. Second, only a small amount of AH could be obtained because of anatomical features, which limited our ability to conduct subsequent validation assays. Third, the development of multiplex immunoassays can be improved. Finally, we can only provide the results of proteomic and ELISA data correlated with smoking and DM risk factors. The exact pathway by which the alpha-2-HS-glycoprotein is involved in cataract pathogenesis remains unclear. More future investigations of molecular pathways are required to discuss how and why the proteomics data varied with smoking and DM, and finally to supply better knowledge of cataracts for the whole of humanity. More studies are also required to analyze the alpha-2-HS-glycoprotein levels in AH of non-diabetic cataract patients, along with further serum and AH comparison analyses of cataract patients with diabetes. In conclusion, our results are from a pioneering exploration of the protein profile for the risk factors involved in cataracts. Cataracts form because of a complicated pathological process involving several proteins that participate in immune reactions and metabolic processes that were identified in AH using a proteomics analysis. The alpha-2-HS-glycoprotein, called fetuin-a, could be a potential aqueous biomarker associated with DM and smoking, which are cataract risk factors. Additional studies are required to complete the analysis and to understand the functions of these cataract-specific proteins, which could provide significant information for the diagnosis, clinical treatment, and prognosis of cataracts.

## Figures and Tables

**Table 1:** Demographic characteristics of enrolled patients with a single risk factor, those with Double risk factors, and cataract controls.

	Cataract Control	Single Risk	Double Risks	*p* Value ^#^
Gender				0.003
Female	7 (77.8%)	3 (30.0%)	0 (0.0%)	
Male	2 (22.2%)	7 (70.0%)	8 (100.0%)	
Protein (μg/μL)	0.22 ± 0.06	0.36 ± 0.21	0.34 ± 0.11	0.049
Age (years)	74.00 ± 5.72	72.30 ± 10.14	69.38 ± 9.87	0.390
VA (logMAR)	0.41 ± 0.12	0.38 ± 0.14	0.27 ± 0.20	0.360
AXL (mm)	23.48 ± 0.59	24.02 ± 1.24	23.69 ± 0.95	0.552
Smoking		5		
Diabetes mellitus (DM)		5		
Smoking + DM			8	

^#^ By Fisher’s exact test, Wilcoxon test, or Kruskal–Wallis test.

**Table 2:** List of aqueous humor (AH) proteins identified by LC-ESI-MS/MS.

Q9NQ66	1-phosphatidylinositol 4,5-bisphosphate phosphodiesterase beta-1	P0CG04	Immunoglobulin lambda constant 1
Q99460	26S proteasome non-ATPase regulatory subunit 1	P01700	Immunoglobulin lambda variable 1–47
O95996	Adenomatous polyposis coli protein 2	P0DOX8	Immunoglobulin lambda-1 light chain
P02768	Albumin	B9A064	Immunoglobulin lambda-like polypeptide 5
P51648	Aldehyde dehydrogenase family 3 member A2	P24592	Insulin-like growth factor-binding protein 6
P02763	Alpha-1-acid glycoprotein 1	Q16270	Insulin-like growth factor-binding protein 7
P19652	Alpha-1-acid glycoprotein 2	Q14624	Inter-alpha-trypsin inhibitor heavy chain H4
P01011	Alpha-1-antichymotrypsin	Q6UXX5	Inter-alpha-trypsin inhibitor heavy chain H6
P01009	Alpha-1-antitrypsin	Q17R60	Interphotoreceptor matrix proteoglycan 1
P04217	Alpha-1B-glycoprotein	Q9BZV3	Interphotoreceptor matrix proteoglycan 2
P02765	Alpha-2-HS-glycoprotein	P01042	Kininogen-1
P01023	Alpha-2-macroglobulin	P02750	Leucine-rich alpha-2-glycoprotein
P02489	Alpha-crystallin A chain	Q68G74	LIM/homeobox protein Lhx8
A0A140G945	Alpha-crystallin A2 chain	P51884	Lumican
P02511	Alpha-crystallin B chain	P61626	Lysozyme C
P06733	Alpha-enolase	P01033	Metalloproteinase inhibitor 1
P03950	Angiogenin	P05408	Neuroendocrine protein 7B2
P01019	Angiotensinogen	P61916	NPC intracellular cholesterol transporter 2
P01008	Antithrombin-III	Q9UBM4	Opticin
P02647	Apolipoprotein A-I	P10451	Osteopontin
P02652	Apolipoprotein A-II	Q9UQ90	Paraplegin
P06727	Apolipoprotein A-IV	P36955	Pigment epithelium-derived factor
P05090	Apolipoprotein D	Q15149	Plectin
P02649	Apolipoprotein E	P0CG47	Polyubiquitin-B
P54253	Ataxin-1	P0CG48	Polyubiquitin-C
P02749	Beta-2-glycoprotein 1	Q9ULS6	Potassium voltage-gated channel subfamily S member 2
P61769	Beta-2-microglobulin	O94913	Pre-mRNA cleavage complex 2 protein Pcf11
P05813	Beta-crystallin A3	Q13395	Probable methyltransferase TARBP1
P53674	Beta-crystallin B1	A0A075B6H7	Probable non-functional immunoglobulin kappa variable 3–7
P43320	Beta-crystallin B2	O94823	Probable phospholipid-transporting ATPase VB
P19022	Cadherin-2	Q9UHG2	ProSAAS
P07339	Cathepsin D	P41222	Prostaglandin-H2 D-isomerase
Q8N163	Cell cycle and apoptosis regulator protein 2	Q92520	Protein FAM3C
Q7Z7A1	Centriolin	P05109	Protein S100-A8
P36222	Chitinase-3-like protein 1	Q9H6Z4	Ran-binding protein 3
Q9HAW4	Claspin	P10745	Retinol-binding protein 3
O43809	Cleavage and polyadenylation specificity factor subunit 5	P02753	Retinol-binding protein 4
P10909	Clusterin	P34096	Ribonuclease 4
P01024	Complement C3	P07998	Ribonuclease pancreatic
P0C0L4	Complement C4-A	Q5T481	RNA-binding protein 20
P0C0L5	Complement C4-B	O75326	Semaphorin-7A
P00751	Complement factor B	P02787	Serotransferrin
P00746	Complement factor D	P00441	Superoxide dismutase [Cu-Zn]
P05156	Complement factor I	P05452	Tetranectin
P01034	Cystatin-C	Q8WZ42	Titin
Q8WVS4	Cytoplasmic dynein 2 intermediate chain 1	O15050	TPR and ankyrin repeat-containing protein 1
Q96M86	Dynein heavy chain domain-containing protein 1	Q15582	Transforming growth factor-beta-induced protein ig-h3
P49792	E3 SUMO-protein ligase RanBP2	Q14956	Transmembrane glycoprotein NMB
Q9HC35	Echinoderm microtubule-associated protein-like 4	P02766	Transthyretin
Q13822	Ectonucleotide pyrophosphatase/phosphodiesterase family member 2	P60174	Triosephosphate isomerase
Q8TE68	Epidermal growth factor receptor kinase substrate 8-like protein 1	P35030	Trypsin-3
P02671	Fibrinogen alpha chain	P62979	Ubiquitin-40S ribosomal protein S27a
Q6ZV73	FYVE, RhoGEF and PH domain-containing protein 6	P62987	Ubiquitin-60S ribosomal protein L40
P07320	Gamma-crystallin D	Q5THJ4	Vacuolar protein sorting-associated protein 13D
P22914	Gamma-crystallin S	P02774	Vitamin D-binding protein
P06396	Gelsolin	Q96PQ0	VPS10 domain-containing receptor SorCS2
P22352	Glutathione peroxidase 3	Q9P202	Whirlin
Q14789	Golgin subfamily B member 1	P25311	Zinc-alpha-2-glycoprotein
P00738	Haptoglobin	P0CG04	Immunoglobulin lambda constant 1
P69905	Hemoglobin subunit alpha	P01700	Immunoglobulin lambda variable 1–47
P68871	Hemoglobin subunit beta	P0DOX8	Immunoglobulin lambda-1 light chain
P02042	Hemoglobin subunit delta	B9A064	Immunoglobulin lambda-like polypeptide 5
P02790	Hemopexin	P24592	Insulin-like growth factor-binding protein 6
P62805	Histone H4	Q16270	Insulin-like growth factor-binding protein 7
P0DOX3	Immunoglobulin delta heavy chain	Q14624	Inter-alpha-trypsin inhibitor heavy chain H4
P0DOX5	Immunoglobulin gamma-1 heavy chain	Q6UXX5	Inter-alpha-trypsin inhibitor heavy chain H6
P01859	Immunoglobulin heavy constant gamma 2	Q17R60	Interphotoreceptor matrix proteoglycan 1
P01860	Immunoglobulin heavy constant gamma 3	Q9BZV3	Interphotoreceptor matrix proteoglycan 2
P01861	Immunoglobulin heavy constant gamma 4	P01042	Kininogen-1
P01780	Immunoglobulin heavy variable 3–7	P02750	Leucine-rich alpha-2-glycoprotein
A0A0B4J1Y9	Immunoglobulin heavy variable 3–72	Q68G74	LIM/homeobox protein Lhx8
A0A0B4J1X5	Immunoglobulin heavy variable 3–74	P51884	Lumican
A0A0J9YXX1	Immunoglobulin heavy variable 5-10-1	P61626	Lysozyme C
A0A0B4J1U7	Immunoglobulin heavy variable 6-1	P01033	Metalloproteinase inhibitor 1
P01834	Immunoglobulin kappa constant	P05408	Neuroendocrine protein 7B2
P0DOX7	Immunoglobulin kappa light chain	P61916	NPC intracellular cholesterol transporter 2
P01624	Immunoglobulin kappa variable 3–15	Q9UBM4	Opticin
P01619	Immunoglobulin kappa variable 3–20	P10451	Osteopontin

**Table 3:** Pathway analysis of aqueous humor (AH) proteins using IPA tools.

Canonical Pathways	Overlap of Proteins in the Single-Risk and Cataract Control Groups	Overlap of Proteins in the Double-Risk and Cataract Control Groups	Overlap of Proteins in the Single- and Double-Risk Groups
LXR/RXR Activation	12	10	1
FXR/RXR Activation	12	10	1
Acute-Phase Response Signaling	11	11	1
Clathrin-mediated Endocytosis Signaling	12		
Atherosclerosis Signaling	7		
Primary Immunodeficiency Signaling		5	
IL-15 Signaling		9	1
B Cell Receptor Signaling			1

Single risk, patients with the DM or smoking risk factor; double risk, patients with both the DM and smoking risk factors; control, cataract patients with neither of these cataract risk factors.

**Table 4:** List of selected potential biomarker candidates.

Protein-ID	Protein Name	Cataract Control (Spc)	Single (Spc)	Multiple of Change (Spc)	Cataract Control (Spc)	Double (Spc)	Multiple of Change (Spc)
Q99460	26S proteasome non-ATPase regulatory subunit 1	0.76 ± 1.18	2.99 ± 0.91	3.93	0.76 ± 1.18	2.95 ± 1.90	3.88
P02763	Alpha-1-acid glycoprotein 1	3.26 ± 3.45	0.00 ± 0.00	0	3.26 ± 3.45	0.00 ± 0.00	0
P19652	Alpha-1-acid glycoprotein 2	2.06 ± 1.89	0.00 ± 0.00	0	2.06 ± 1.89	0.00 ± 0.00	0
P01011	Alpha-1-antichymotrypsin	2.87 ± 2.07	0.32 ± 0.52	0.11	2.87 ± 2.07	0.26 ± 0.74	0.09
P02765	Alpha-2-HS-glycoprotein	0.00 ± 0.00	2.14 ± 1.72	−100	0.00 ± 0.00	4.30 ± 2.08	−100
P02647	Apolipoprotein A-I	3.88 ± 4.11	10.49 ± 2.19	2.68	3.88 ± 4.11	9.41 ± 6.49	2.43
P02652	Apolipoprotein A-II	0.09 ± 0.26	2.09 ± 1.33	23.22	0.09 ± 0.26	2.26 ± 1.52	25.11
P02749	Beta-2-glycoprotein 1	1.90 ± 1.49	0.09 ± 0.27	0.05	1.90 ± 1.49	0.33 ± 0.63	0.17
P36222	Chitinase-3-like protein 1	5.39 ± 2.93	1.15 ± 1.87	0.21	5.39 ± 2.93	0.71 ± 0.88	0.13
Q13822	Ectonucleotide pyrophosphatase/phosphodiesterase family member 2	3.63 ± 3.78	0.11 ± 0.34	0.03	3.63 ± 3.78	0.14 ± 0.41	0.04
P22352	Glutathione peroxidase 3	1.15 ± 1.23	0.00 ± 0.00	0	1.15 ± 1.23	0.00 ± 0.00	0
Q14789	Golgin subfamily B member 1	0.54 ± 0.71	0.00 ± 0.00	0	0.54 ± 0.71	0.00 ± 0.00	0
P02790	Hemopexin	21.12 ± 8.44	1.56 ± 1.62	0.07	21.12 ± 8.44	2.67 ± 3.40	0.13
P0DOX5	Immunoglobulin gamma-1 heavy chain	34.76 ± 6.08	10.24 ± 4.37	0.29	34.76 ± 6.08	10.58 ± 5.89	0.3
P01859	Immunoglobulin heavy constant gamma 2	21.29 ± 3.52	5.29 ± 3.57	0.25	21.29 ± 3.52	6.27 ± 4.97	0.3
P01860	Immunoglobulin heavy constant gamma 3	22.01 ± 4.99	6.75 ± 3.30	0.31	22.01 ± 4.99	6.98 ± 4.25	0.32
P01861	Immunoglobulin heavy constant gamma 4	15.02 ± 3.42	4.14 ± 2.88	0.28	15.02 ± 3.42	4.92 ± 2.49	0.33
P01780	Immunoglobulin heavy variable 3–7	2.46 ± 2.13	0.00 ± 0.00	0	2.46 ± 2.13	0.25 ± 0.72	0.1
A0A0B4J1Y9	Immunoglobulin heavy variable 3–72	1.67 ± 1.09	0.00 ± 0.00	0	1.67 ± 1.09	0.13 ± 0.36	0.08
A0A0B4J1X5	Immunoglobulin heavy variable 3–74	2.08 ± 1.99	0.00 ± 0.00	0	2.08 ± 1.99	0.25 ± 0.72	0.12
A0A0B4J1U7	Immunoglobulin heavy variable 6–1	1.16 ± 1.28	0.09 ± 0.27	0.08	1.16 ± 1.28	0.00 ± 0.00	0
P01834	Immunoglobulin kappa constant	16.50 ± 5.02	2.75 ± 2.24	0.17	16.50 ± 5.02	2.94 ± 2.81	0.18
P0DOX7	Immunoglobulin kappa light chain	12.23 ± 3.03	2.75 ± 2.24	0.23	12.23 ± 3.03	2.94 ± 2.81	0.24
P0CG04	Immunoglobulin lambda constant 1	4.74 ± 1.71	2.29 ± 1.75	0.48	4.74 ± 1.71	1.66 ± 1.20	0.35
P0DOX8	Immunoglobulin lambda-1 light chain	4.74 ± 1.71	2.29 ± 1.75	0.48	4.74 ± 1.71	1.66 ± 1.20	0.35
B9A064	Immunoglobulin lambda-like polypeptide 5	4.74 ± 1.71	2.29 ± 1.75	0.48	4.74 ± 1.71	1.66 ± 1.20	0.35
Q16270	Insulin-like growth factor-binding protein 7	3.52 ± 1.34	1.83 ± 1.03	0.52	3.52 ± 1.34	1.09 ± 1.28	0.31
P01033	Metalloproteinase inhibitor 1	0.78 ± 0.80	0.00 ± 0.00	0	0.78 ± 0.80	0.00 ± 0.00	0
P61916	NPC intracellular cholesterol transporter 2	1.05 ± 0.89	0.00 ± 0.00	0	1.05 ± 0.89	0.20 ± 0.58	0.19
Q92520	Protein FAM3C	1.50 ± 1.23	0.00 ± 0.00	0	1.50 ± 1.23	0.00 ± 0.00	0
P02753	Retinol-binding protein 4	2.09 ± 0.97	0.71 ± 1.30	0.34	2.09 ± 0.97	0.86 ± 0.96	0.41
O75326	Semaphorin-7A	0.98 ± 1.59	0.00 ± 0.00	0	0.98 ± 1.59	0.00 ± 0.00	0
P02787	Serotransferrin	74.79 ± 23.85	31.40 ± 9.50	0.42	74.79 ± 23.85	30.22 ± 9.85	0.4
P00441	Superoxide dismutase [Cu-Zn]	2.93 ± 1.87	0.19 ± 0.41	0.06	2.93 ± 1.87	0.25 ± 0.72	0.09
P05452	Tetranectin	2.53 ± 1.41	0.00 ± 0.00	0	2.53 ± 1.41	0.00 ± 0.00	0
P25311	Zinc-alpha-2-glycoprotein	8.92 ± 2.57	0.00 ± 0.00	0	8.92 ± 2.57	0.52 ± 1.12	0.06
P06727	Apolipoprotein A-IV	0.11 ± 0.32	5.51 ± 4.11	50.09			
P02649	Apolipoprotein E	1.04 ± 1.80	3.92 ± 2.74	3.77			
O43809	Cleavage and polyadenylation specificity factor subunit 5	0.98 ± 0.68	0.21 ± 0.68	0.21			
P01619	Immunoglobulin kappa variable 3–20	1.06 ± 1.34	0.00 ± 0.00	0			
P24592	Insulin-like growth factor-binding protein 6	1.88 ± 1.31	0.23 ± 0.72	0.12			
Q9UBM4	Opticin	0.09 ± 0.26	0.64 ± 0.74	7.11			
P0CG47	Polyubiquitin-B	1.54 ± 1.41	0.10 ± 0.33	0.06			
P0CG48	Polyubiquitin-C	1.54 ± 1.41	0.10 ± 0.33	0.06			
Q9ULS6	Potassium voltage-gated channel subfamily S member 2	0.11 ± 0.32	0.65 ± 0.75	5.91			
P62979	Ubiquitin-40S ribosomal protein S27a	1.54 ± 1.41	0.10 ± 0.33	0.06			
P62987	Ubiquitin-60S ribosomal protein L40	1.54 ± 1.41	0.10 ± 0.33	0.06			
P61769	Beta-2-microglobulin				5.22 ± 2.45	2.02 ± 1.83	0.39
P0C0L4	Complement C4-A				0.41 ± 0.82	2.16 ± 2.53	5.27
P0C0L5	Complement C4-B				0.41 ± 0.82	2.16 ± 2.53	5.27
P41222	Prostaglandin-H2 D-isomerase				11.39 ± 1.97	8.00 ± 1.65	0.71

Single risk, patients with the DM or smoking risk factor; double risk, patients with both the DM and smoking risk factors; control, cataract patients with neither of these cataract risk factors; Spc, spectral count.

## Data Availability

Not applicable.
